# Genetic Analysis of Fusarium Head Blight Resistance in CIMMYT Bread Wheat Line C615 Using Traditional and Conditional QTL Mapping

**DOI:** 10.3389/fpls.2018.00573

**Published:** 2018-05-01

**Authors:** Xin Yi, Jingye Cheng, Zhengning Jiang, Wenjing Hu, Tongde Bie, Derong Gao, Dongsheng Li, Ronglin Wu, Yuling Li, Shulin Chen, Xiaoming Cheng, Jian Liu, Yong Zhang, Shunhe Cheng

**Affiliations:** ^1^Key Laboratory of Wheat Biology and Genetic Improvement for Low & Middle Yangtze Valley Ministry of Agriculture, Lixiahe Agricultural Institute of Jiangsu Province, Yangzhou, China; ^2^National Key Laboratory of Crop Genetics and Germplasm Enhancement, Nanjing Agricultural University, Nanjing, China; ^3^College of Agronomy, Yangzhou University, Yangzhou, China; ^4^College of Agronomy, Henan Agricultural University, Zhengzhou, China

**Keywords:** conditional QTL analysis, Fusarium head blight, SNP marker, traditional QTL analysis, *Triticum aestivum*

## Abstract

Fusarium head blight (FHB) is a destructive wheat disease present throughout the world, and host resistance is an effective and economical strategy used to control FHB. Lack of adequate resistance resource is still a main bottleneck for FHB genetics and wheat breeding research. The synthetic-derived bread wheat line C615, which does not carry the *Fhb1* gene, is a promising source of FHB resistance for breeding. A population of 198 recombinant inbred lines (RILs) produced by crossing C615 with the susceptible cultivar Yangmai 13 was evaluated for FHB response using point and spray inoculations. As the disease phenotype is frequently complicated by other agronomic traits, we used both traditional and multivariate conditional QTL mapping approaches to investigate the genetic relationships (at the individual QTL level) between FHB resistance and plant height (PH), spike compactness (SC), and days to flowering (FD). A linkage map was constructed from 3,901 polymorphic SNP markers, which covered 2,549.2 cM. Traditional and conditional QTL mapping analyses found 13 and 22 QTL for FHB, respectively; 10 were identified by both methods. Among these 10, three QTL from C615 were detected in multiple years; these QTL were located on chromosomes 2AL, 2DS, and 2DL. Conditional QTL mapping analysis indicated that, at the QTL level, SC strongly influenced FHB in point inoculation; whereas PH and SC contributed more to FHB than did FD in spray inoculation. The three stable QTL (*QFhbs-jaas.2AL, QFhbp-jaas.2DS*, and *QFhbp-jaas.2DL*) for FHB were partly affected by or were independent of the three agronomic traits. The QTL detected in this study improve our understanding of the genetic relationships between FHB response and related traits at the QTL level and provide useful information for marker-assisted selection for the improvement of FHB resistance in breeding.

## Introduction

Fusarium head blight (FHB), also known as head scab, is one of the most destructive fungal diseases of wheat and is found in temperate and subtropical regions around the world. The most important fungal pathogens associated with FHB are *Fusarium graminearum* and *Fusarium culmorum* (Parry et al., 1995). FHB reduces grain yield and quality due to shriveled kernels with low weight and contamination of several mycotoxins, such as deoxynivalenol (DON). Infected kernels with excessive DON pose a severe threat to human and animal health (McMullen et al., 2012). In 2015, the Codex Alimentarius Commission (CAC) enacted standards regulating DON content, with maximum permitted levels of 2 ppm and 1 ppm for unprocessed and finished wheat products, respectively, and 0.2 ppm for baby food (Codex Alimentarius Commission [CAC], 2015). FHB outbreaks have become more serious and more frequent in recent decades, possibly due to climate change and agronomic practices (especially maize–wheat rotations). Globally, FHB causes about 10–70% yield loss in epidemic years (Zhang et al., 2011). In the Middle-Lower Yangtze River Valley Region of China, FHB causes 5–10% yield loss in most years but can cause losses of nearly 100% in severe epidemic years (Cheng et al., 2012). Host resistance is currently recognized as the most effective and environmentally friendly method of FHB management (He et al., 2013), justifying the use of resistant varieties to control FHB.

The genetics of FHB resistance are complex since this trait is under multigenic control and is subject to genotype × environment interactions. Five types of FHB resistance mechanisms have been described (Mesterhazy et al., 1999): Type I (resistance to initial infection), Type II (resistance to disease spread within infected heads), Type III (resistance to DON accumulation), Type IV (resistance to kernel damage), and Type V (tolerance). More than 250 FHB QTL have been reported, covering all 21 wheat chromosomes (Buerstmayr et al., 2009; Liu et al., 2009). Among these, *Fhb1* from the Chinese variety Sumai 3 is the resistance gene most often used by breeding programs to combat FHB (Cuthbert et al., 2006; Lv et al., 2014). *Fhb2* from Sumai 3 (Yang et al., 2006; Lu et al., 2010) and *Fhb7* (Guo et al., 2015) from *Thinopyrum ponticum* have also been used by resistance breeding programs. However, it is still a formidable challenge to transfer resistance genes into susceptible cultivars because major sources of resistance (such as Sumai 3 and Wangshuibai) also carry undesirable agronomic traits (Dvorjak, 2014; Li et al., 2016).

Several studies have showed that agronomic traits may be associated with FHB resistance. Plant height (PH) and days to flowering (FD) are often negatively correlated to FHB severity (Mao et al., 2010; Lu et al., 2011; Buerstmayr et al., 2012; Giancaspro et al., 2016; McCartney et al., 2016). Positive correlations between spike compactness (SC) and FHB severity have also been commonly reported (Buerstmayr et al., 2011; Giancaspro et al., 2016). Other traits associated with FHB include heading time (Emrich et al., 2008), degree of anther extrusion (Skinnes et al., 2010; Lu et al., 2013), and presence/absence of awns (Ban and Suenaga, 2000). Among these reports, researchers have generally analyzed the genetic relationship between FHB response and agronomic traits using QTL mapping approaches that first ask whether QTL are closely linked or pleiotropic. However, traditional QTL mapping analysis of genetic relationships of complex traits is often confounded with variations involving other traits (Cui et al., 2012; Li et al., 2015). Multivariable conditional analysis has been utilized in evaluating the conditional phenotypic values of a target trait by excluding the effects of related traits as well as in determining the contribution of related traits to a target trait. By comparatively analyzing both conditional and unconditional QTL, we may be able to identify the genetic relationships among different traits at the QTL level (Zhu, 1995; Wen and Zhu, 2005). Thus far, various studies on wheat using this methodology have examined PH (Cui et al., 2011; Yu et al., 2014), kernel weight (Li et al., 2015), yield (Xu et al., 2014; Fan et al., 2015), and seedling traits (Zhang et al., 2014). Such studies indicate that conditional QTL analysis is helpful for understanding the influence of one trait on another complex trait at the QTL level. However, conditional QTL mapping analyses of the genetic relationships between FHB response and agronomic traits is limited.

The identification of new FHB-resistant germplasms has broadened the resistance gene pool as well as facilitated the improvement of resistance in cultivars, as wheat breeding has thus far relied heavily on Sumai 3 and its derivatives, with *Fhb1* as donor parents. Several synthetic hexaploid wheat (SHW) lines and their derivatives developed by the International Maize and Wheat Improvement Center (CIMMYT) have been used in resistance breeding programs around the world (He et al., 2013, 2016; Zhu et al., 2016). The SHW-derived CIMMYT line C615 exhibits moderate resistance to FHB in the field, but haplotype analysis revealed that it appeared to lack *Fhb1*. In this study, a recombinant inbred line (RIL) population was developed from a cross between C615 and a susceptible parent, Yangmai 13. The objectives of the study were to (1) dissect the FHB resistance QTL using a high-density SNP map; (2) identify SNP markers closely linked to resistance loci; and (3) analyze the genetic relationships between FHB response and morphological traits and FD at the QTL level using multivariable conditional QTL mapping analysis.

## Materials and Methods

### Plant Materials

C615 is a CIMMYT synthetic derived line (kindly provided by Prof. A. Mujeeb-kazi, CIMMYT, Mexico) with moderate FHB resistance and good adaptability at Yangzhou. It has the pedigree SABUF/3/BCN//CETA/AE.SQUARROSA (895), where SABUF and BCN are CIMMYT bread wheat genotypes, and CETA is a durum variety. Yangmai 13 is a FHB-susceptible Chinese soft wheat cultivar and has been widely planted in the Middle-Lower Yangtze River region. One hundred and ninety eight F_7_ RILs were produced by single seed descent from cross C615/Yangmai 13. For FHB evaluation, Sumai 3, Yangmai 158, and Annong 8455 were used as resistant, moderately resistant and susceptible controls, respectively.

### Phenotypic Evaluation

The RILs and parents (C615 and Yangmai 13) were evaluated for FHB response and related agronomic traits at Lixiahe Agricultural Institute of Jiangsu Province, Yangzhou, during 2014–2015 (E1), 2015–2016 (E2), and 2016–2017 (E3). Field experiments were designed as randomized complete blocks with two replicates per environment. The RILs in each replication were sown in two 133 cm rows with 40 seeds per row, with a row spacing of 25 cm. The field trials were managed following local practices.

#### Point Inoculation

All materials were point inoculated with four *F. graminearum* strains (F4, F15, F34, and F0609) kindly provided by Prof. Huaigu Chen, Jiangsu Academy of Agricultural Sciences. Inoculations were performed at the late heading stage when 5 μl of macroconidial suspension (1.0 × 10^5^ conidia/ml) was injected into a single floret in the middle of each spike; 30 spikes were inoculated per row (Lu et al., 2011). After inoculation, the disease nursery was mist-irrigated for 5 min every half-hour from 7:00 am to 6:00 pm each day to provide high humid conditions favorable for FHB infection. FHB severities were recorded 20 days after inoculation as the number of symptomatic spikelets per infected spike, and mean data were used for analysis.

#### Spray Inoculation

The two-replicate field nursery for spray inoculation at flowering were grown with two replicates for 2 years (E2 and E3). Central plants in each plot were inoculated by spraying a mixed macroconidial suspension (same as for point inoculation) with a backpack sprayer and was repeated 2–3 days later (He et al., 2016). The same misting system as for point-inoculated experiments was applied. FHB severity was calculated by recording the percentage of symptomatic spikelets of 30 spikes per plot (Lu et al., 2013).

The RILs and parents were also evaluated for three agronomic traits reported to be related to FHB response, i.e., PH, SC, and FD over 3 years (E1, E2, and E3). PH was measured from the ground to the top of the spikes excluding awns. SC was calculated from the spike length (SL) and spikelet number per spike (SNS) according to the equation: SC = SNS/SL (Lv et al., 2014). PH and SC were recorded as average values of twenty individual plants per line. FD was recorded when 50% of spikes a line were at anthesis.

### Genotyping and Linkage Map Construction

Genomic DNA for SNP assays was extracted from young leaf tissues by the CTAB method (Stacey and Isaac, 1994). The 198 RILs and two parents were genotyped using the wheat 90K iSelect array with 81,587 SNP (Wang et al., 2014). Genotyping assays were carried out on the Illumina iScan reader and made genotypic clusters for each SNP using GenomeStudio software 1.9.4 (Illumina; http://www.illumina.com).

Prior to mapping, SNP data were evaluated following Zhai et al. (2015). SNPs with more than 20% missing values or strong segregation distortion were excluded from linkage mapping. Linkage groups were constructed using Joinmap V4.0 (Stam, 1993) with a minimum independent logarithm of odds (LOD) threshold of 10.0. The Kosambi mapping function was used to estimate genetic distances (in cM) between markers, with a maximum recombination threshold of 0.4 and a jump threshold of 5.0 (Kosambi, 1943). Linkage maps were generated with the software MapChart 2.2^1^ (Voorrips, 2002). The long (L) and short (S) arms of each chromosome were identified from the wheat 90K consensus SNP map (Wang et al., 2014).

### Data Analysis and QTL Mapping

Analysis of variance (ANOVA) and phenotypic correlation coefficients were conducted using SAS v.9.2 software (SAS Institute Inc., Cary, NC, United States). Broad-sense heritability (*h^2^*) of each trait was calculated using *h^2^* = σ^2^*_g_*/[σ^2^*_g_* + σ^2^*_gy_*/*r* + σ^2^*_e_*/*ry*] for multiple years, where σ^2^*_g_* is the estimate of genetic variance, σ^2^*_gy_* is the estimate of genotype × year interaction variance, σ^2^*_e_* is the estimate of residual error variance, *r* is the number of replicates per line, and *y* is the number of years.

Both conditional and traditional QTL analyses were conducted using composite interval mapping (CIM) using QTL Cartographer 2.5^2^ (Wang et al., 2006). The LOD value was set at 3.0 after 1,000 permutations to declare of putative QTL. The QTL (2.5 < LOD < 3.0) were also reported for other environments when these detected in at least one environment reaching the significance level. QTL intervals were estimated that a 2-LOD fell off support interval with a confidence threshold 95% (van Ooijen, 1992).

Unconditional and conditional QTL were estimated based on phenotypic and conditional phenotypic values of the traits, respectively. Conditional phenotypic values of y_(FHBjFRATs)_ were obtained from QGAStation 2.0^3^ as described by Zhu (1995) and were estimated through using a two-step procedure described by Cui et al. (2012); here, FHB|FRATs refers to FHB conditioned on FHB-related agronomic traits (FRATs; e.g., FHB|PH means FHB conditioned on PH). The QTL data menu settings of QGAStation 2.0 were implemented based on a method described by Cui et al. (2012).

QTL were named as follows: *Qtrait-lab designation.chromosome location-X*, a number distinguishing multiple linkage groups (LGs) within the same chromosome. QTL for FHB resistance identified by point inoculation were designed as *QFhbp*- whereas *QFhbs*- was used for those identified by spray inoculation. For example, *QFhbp-jaas.2B-2* indicates an FHB resistance QTL on the second LGs of chromosome 2B that was identified following point inoculation.

## Results

### Phenotypic Evaluation and Correlations Between FHB and Related Traits

**FIGURE 1:**
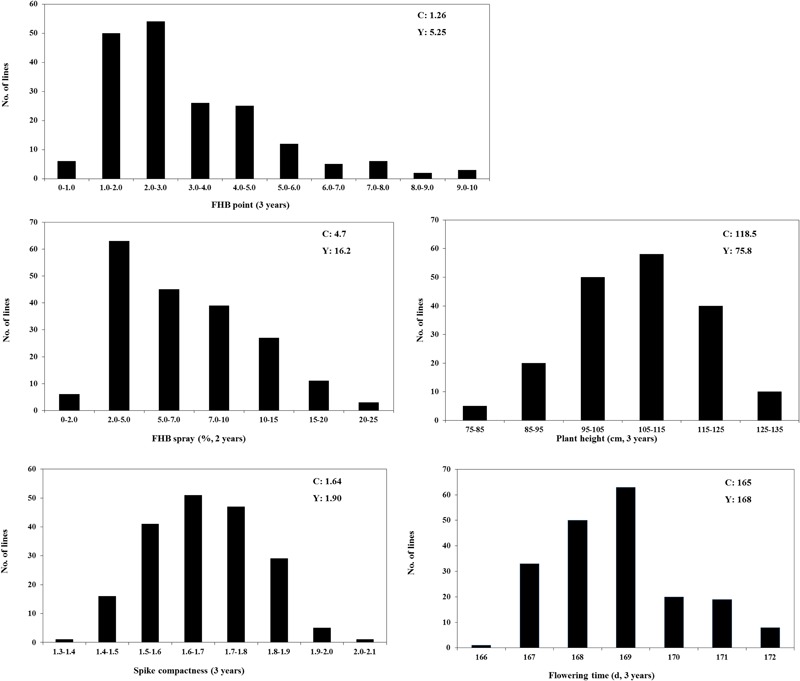
Frequency distributions for FHB, Plant height, Spike compactness, and Days to Flowering in the C615/Yangmai 13 RIL population based on the mean data across environments. C: C615, Y: Yangmai 13.

The mean phenotypic values, RIL range, parental response, and broad-sense heritability for each trait are presented in **Table 1**. C615 was taller than Yangmai 13, whereas the latter had higher FHB severity, SC, and FD in all environments. The broad-sense heritabilities (*h^2^*) based on RIL mean data were 0.87 for point inoculation, 0.78 for spray inoculation, 0.97 for PH, 0.77 for SC, and 0.88 for FD. Large variations among RILs were observed for all traits. Based on the mean data across environments, the frequency distributions of each trait for the RILs were continuous and strong transgressive segregation was evident (**Figure 1**).

**Table 1:** Phenotypic data and broad-sense heritabilities for FHB severity and correlated traits in C615/Yangmai 13 RILs and parents across environments.

Trait^a^	Environment^b^	Parent		RILs		
		C615	Yangmai 13	Mean ± SD	Range	Heritability
FHB point						0.87
	YZ 2015	1.01	4.55	3.65 ± 1.92	0.56-8.80	
	YZ 2016	1.16	5.61	2.16 ± 2.08	0.62-11.00	
	YZ 2017	1.61	5.59	3.47 ± 2.22	0.48-11.54	
	Mean	1.26	5.25	3.22 ± 1.93	0.58-9.58	
FHB spray (%)						0.78
	YZ 2016	6.15	21.68	8.61 ± 6.18	1.30-33.56	
	YZ 2017	3.22	10.79	4.90 ± 3.16	0.24-22.45	
	Mean	4.69	16.24	6.81 ± 4.53	0.92-24.41	
PH (cm)						0.97
	YZ 2015	116.3	74.3	104.6 ± 9.9	82.6-131.1	
	YZ 2016	120.5	74.5	105.6 ± 12.3	75.8-138.1	
	YZ 2017	118.7	78.7	110.0 ± 12.4	80.3-143.3	
	Mean	118.5	75.8	106.7 ± 10.9	81.8-133.6	
SC						0.77
	YZ 2015	1.62	1.88	1.68 ± 0.13	1.36-2.04	
	YZ 2016	1.65	1.95	1.70 ± 0.16	1.33-2.10	
	YZ 2017	1.66	1.86	1.66 ± 0.14	1.35-2.05	
	Mean	1.64	1.90	1.68 ± 0.13	1.40-2.06	
FD (d)						0.88
	YZ 2015	167.0	170.0	175.1 ± 1.8	168.0-176.0	
	YZ 2016	169.0	171.0	168.7 ± 1.0	167.0-171.0	
	YZ 2017	157.0	165.0	162.7 ± 1.2	161.0-166.0	
	Mean	165.0	168.0	168.9 ± 1.1	166.0-172.0	

*^*a*^FHB point: FHB evaluated after point inoculation; FHB spray: FHB evaluated after spray inoculation; PH: Plant height; SC: Spike compactness; FD: Days to flowering. ^*b*^YZ: Yangzhou.*

Several response variables, including FHB in both the point and spray inoculated experiments, as well as PH, SC, and FD significantly differed (*P* < 0.001) among genotypes, G × E (genotype × year) interactions, and years (environments), except G × E in SC (Supplementary Table S1). After spray inoculation, negative correlations between FHB severity and PH (*r* = -0.37, *P* < 0.01) and FD (*r* = -0.18 to -0.20, *P* < 0.05) were found to be significant; in contrast, point-inoculated experiments found non-significant correlations between FHB severity and PH or FD. FHB severity also showed significant positive correlations with SC (*r* = 0.15 to 0.31, *P* < 0.05) in both types of inoculated experiments. Finally, FHB and related agronomic traits showed weak correlations (i.e., *r* < 0.5) in both the point or spray inoculated experiments (**Table 2**).

**Table 2:** Correlation coefficients between FHB severity and associated agronomic traits in the RIL population derived from C615/Yangmai 13.

	PH	SC	FD
FHB point			
YZ 2015	-0.13	0.15^∗∗^	-0.14
YZ 2016	-0.17	0.21^∗∗^	-0.16
YZ 2017	-0.11	0.31^∗∗^	-0.12
FHB spray			
YZ 2016	-0.37^∗∗^	0.23^∗∗^	-0.18^∗^
YZ 2017	-0.37^∗∗^	0.24^∗∗^	-0.20^∗^

*^∗^, ^∗∗^ Significance at *P* < 0.05, *P* < 0.01 levels, respectively. See footnote to **Table 1** for abbreviations.*

### SNP Genotyping and Linkage Map

Among the 81,587 identified SNPs from the Illumina wheat 90K SNP chip, 6,321 SNPs (7.8%) were polymorphic between the two parents. Among these polymorphic markers, those that were unanchored or linked in small linkage groups (LGs, usually smaller than 5 cM) were excluded from further analyses. A final panel of 3,901 SNPs was used for the construction of a linkage map and for QTL mapping.

Detailed information for the linkage map is provided in Supplementary Tables S2, S3, and the linkage map showed good synteny with the recently released wheat reference genome, with the exception of chromosome 4B, 5A, and 7B (Supplementary Figure S1). The entire linkage map consisted of 34 LGs representing all 21 wheat chromosomes. Chromosomes 1D, 2B, 2D, 3D, 4A, 5A, 5B, 5D, 6D, 7A, and 7B were each assembled into two LGs, and chromosome 6A consisted of three LGs. The overall linkage map covered 2,549.2 cM, with chromosome length ranging from 30.7 cM (4D) to 179.9 cM (4A) with a mean of 121.4 cM. The A genome had 1,810 SNPs (46.4%), with a total length of 1,012.6 cM, and an SNP density of 1.79 markers/cM; the B genome included 1,441 SNPs (36.9%) covering 924.3 cM, and an SNP density of 1.56 markers/cM; and the D genome had 650 SNPs (16.7%) with a total length of 612.3 cM and an SNP density of 1.06 markers/cM. The number of SNP markers on each chromosome ranged from 6 (6B) to 582 (5B) and mean overall SNP density was 1.53 markers/cM, ranging from 0.07 (6B) to 3.64 (5B).

### Traditional QTL Mapping

**FIGURE 2:**
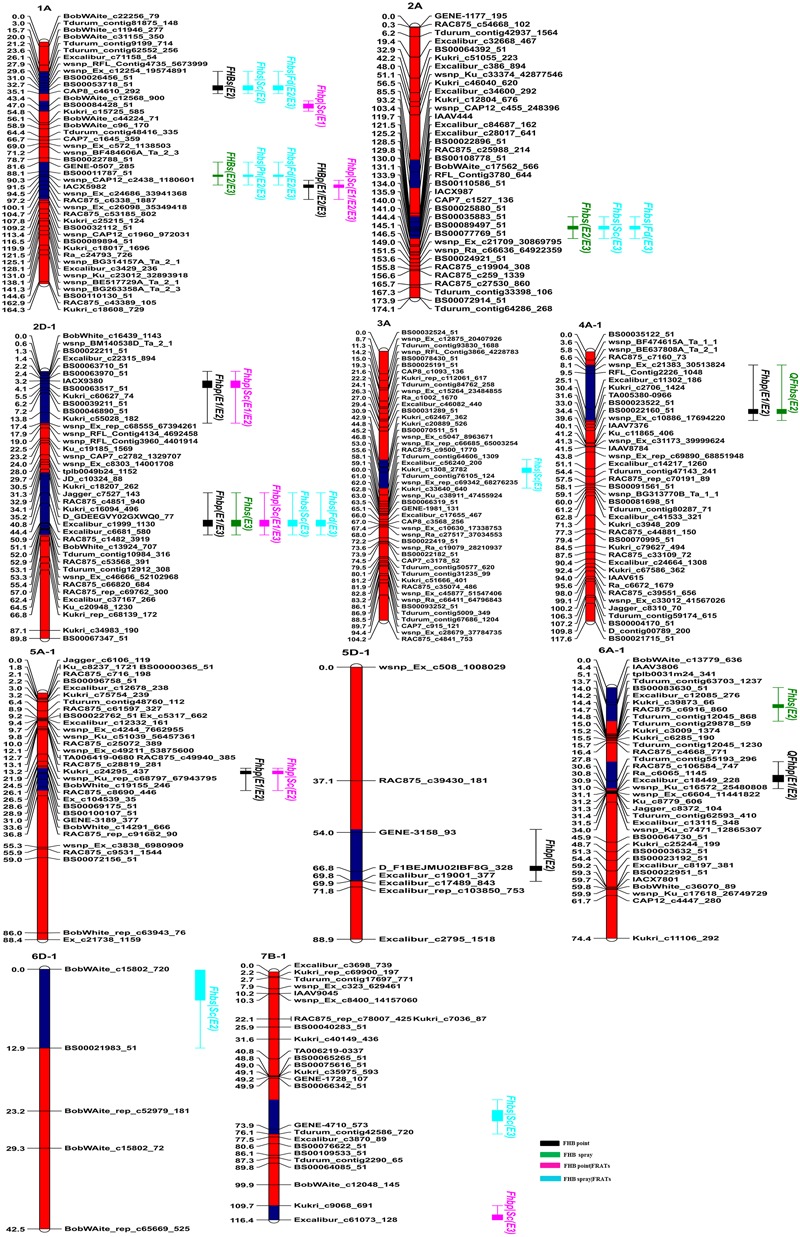
Genetic linkage maps and locations of QTL for FHB resistance in the C615/Yangmai 13 population. Red and blue regions on chromosomes indicate confidence intervals of QTL. QTL names are on the right with different colors for different traits. FHB point, FHB evaluated after point inoculation; FHB spray, FHB evaluated after spray inoculation; FHB point|FRATs, FHB point without the influence of related agronomic traits (e.g., PH, SC, and FD); FHB spray|FRATs, FHB spray without the influence of three related agronomic traits.

In the point-inoculated experiments, seven traditional QTL for FHB resistance were identified on chromosomes 1A, 2D (2), 4A, 5A, 5D, and 6A (**Table 3** and **Figure 2**). A stable QTL on chromosome 2DL (LG, 2D-1, closest marker: *TA001163-0861*) was detected in all 3 years, with LOD values ranging from 4.80 to 6.39 that explained up to 11.79% of the phenotypic variation. *QFhbp-jaas.1AL* (closest marker: *RAC875_c6338_1887*) was detected in 3 years and accounted for 4.83–10.83% of the phenotypic variation. Four QTL identified in 2 years accounted for 4.86–9.28% of the phenotypic variation and were located on chromosomes 2DS, 4AL, 5AL, and 6AS, respectively. The remaining QTL on chromosome 5DL were detected only in 1 year and explained 7.29% of the phenotypic variation. Alleles increasing FHB resistance from C615 were found at four loci (2DS, 2DL, 4AL, and 5DL), and resistance was also conferred by QTL on chromosomes 1AL, 1BL, 5AL, and 6AS that were derived from the susceptible parent, Yangmai 13.

**Table 3:** Composite interval mapping for FHB resistance and agronomic traits in the C615/Yangmai 13 RIL population.

Trait	QTL	Environment^a^	Closest marker^b^	Position interval (cM)	LOD value^c^	PVE (%)^d^	Additive effect^e^
FHB point							
	*QFhbp-jaas.1AL*	E1/E2/E3/ME	RAC875_c6338_1887	94.5-107.8	2.87/4.26/3.39/4.88	4.83/7.31/5.13/10.83	0.42/0.57/0.51/0.67
	*QFhbp-jaas.2DS-1*	E1/E2/ME	Kukri_c60627_74	0.0-17.4	3.80/3.20/2.70	5.43/5.12/4.99	-0.57/-0.52/-0.39
	*QFhbp-jaas.2DL-1*	E1/E2/E3/ME	TA001163-0861	40.8-55.0	5.70/4.80/6.39/6.21	10.16/8.26/11.79/11.36	-0.67/-0.53/-0.80/-0.70
	*QFhbp-jaas.4AL-1*	E1/E2/ME	BS00041735_51	5.8-30.0	2.70/3.50/3.80	4.86/6.12/6.94	-0.42/-0.54/-0.59
	*QFhbp-jaas.5AL-1*	E1/E2/ME	BS00069175_51	26.5-36.8	3.20/4.00/4.90	6.12/6.98/8.69	0.42/0.50/0.57
	*QFhbp-jaas.5DL-1*	E2	D_F1BEJMU02IBF8G_328	53.0-69.8	3.84	7.29	-0.63
	*QFhbp-jaas.6AS-1*	E1/E2	Tdurum_contig55193_296	22.0-30.6	2.60/5.89	4.26/9.28	0.42/0.58
FHB spray							
	*QFhbs-jaas.1AS*	E2/ME	BS00026456_51	20.0-35.1	4.70/5.10	9.56/7.54	-2.15/-1.79
	*QFhbs-jaas.1AL*	E2/E3/ME	wsnp_CAP12_c2438_1180601	81.6-97.2	4.25/3.52/3.05	7.54/6.32/5.43	2.36/1.52/1.24
	*QFhbs-jaas.2AL*	E2/E3/ME	BS00022896_51	121.5-135.9	2.52/3.41/2.80	5.23/9.34/6.13	-1.22/-2.00/-1.45
	*QFhbs-jaas.2DL-1*	E3	TA001163-0861	40.8-55.0	5.20	10.34	-1.97
	*QFhbs-jaas.4AL-1*	E2/ME	BS00041735_51	5.8-30.0	3.20/4.30	5.65/9.61	-1.85/-2.45
	*QFhbs-jaas.6AS-1*	E2	tplb0031m24_341	0.0-10.0	4.05	7.33	-2.44

*^*a*^E1: Yangzhou 2015, E2: Yangzhou 2016, E3: Yangzhou 2017, ME: Pooled data for environments. ^*b*^The closest linked marker to the peak of the LOD contours. ^*c*^A LOD threshold of 3.0 was used for the declaration of a QTL. ^*d*^Percentage of phenotypic variance that could be explained by the QTL. ^*e*^Additive effect with positive and negative values indicating an increasing effect from C615 or Yangmai 13 alleles, respectively.*

In the spray-inoculated experiments, six traditional QTL for FHB resistance were identified on chromosomes 1A (2), 2A, 2D, 4A, and 6A (**Table 3** and **Figure 2**). *QFhbs-jaas.1AL* (closest marker: *wsnp_CAP12_c2438_1180601*) and *QFhbs-2AL* (closest marker: *BS00022896_51*) were detected in 2 years, accounting for 6.32–7.54% and 5.23–9.34% of the phenotypic variance, respectively. The remaining four QTL were identified in 1 year and explained 5.65–10.34% of the phenotypic variance. Alleles for FHB resistance were derived from C615 for all QTL except for the one on chromosome 1AL, where Yangmai 13 contributed the resistance allele. The QTL on chromosomes 1AL, 2DL, and 4AL detected in the spray-inoculated experiments were located within the same chromosomal regions as those detected in the point-inoculated experiments.

### Conditional QTL Mapping

After point inoculation, FHB severity only exhibited a significant correlation with SC (**Table 2**), and three of seven putative traditional QTL for FHB were mapped using conditional QTL analysis (**Table 4**). *QFhbp|Sc-jaas.1AL* showed increased contributions to those of the corresponding traditional QTL in 3 years; Moreover, *QFhbp|Sc-jaas.2DS-1* and *QFhbp|Sc-jaas.2DL-1* showed increased or equal contributions to those of the corresponding traditional QTL in 3 years, and explained 9.78–11.80% and 8.52–11.65% of the phenotypic variations, respectively.

**Table 4:** Genetic analysis of FHB and associated traits at the individual QTL level in the C615/Yangmai 13 RIL population.

Trait	Traditional QTL	Conditional QTL [A(E/PVE%)]^a^		
		FHB|PH	FHB|SC	FHB|FD
FHB point				
	*QFhbp-jaas.1AL*		0.45 (E1/5.82) +	
			0.58 (E2/8.46) +	
			0.55 (E3/6.29) +	
	*QFhbp-jaas.2DS-1*		-0.55 (E1/7.20) +	
			-0.52 (E2/6.88) +	
	*QFhbp-jaas.2DL-1*		-0.78 (E1/11.22) +	
			-0.63 (E1/10.12) +	
			-0.72 (E3/10.81) =	
	*QFhbp-jaas.4AL-1*			
	*QFhbp-jaas.5AL-1*			
	*QFhbp-jaas.5DL-1*			
	*QFhbp-jaas.6AS-1*			
FHB spray	*QFhbs-jaas.1AS*			-2.06 (E2/7.53) –
		-1.33 (E3/4.36) b		-1.57 (E3/5.68) b
	*QFhbs-jaas.1AL*	1.41 (E2/5.14) –	1.52 (E2/5.80) –	1.56 (E2/6.41) –
		1.35 (E3/6.35) =		1.01 (E3/5.43) –
	*QFhbs-jaas.2AL*			
			-0.95 (E3/4.86) –	-2.06 (E3/9.00) =
	*QFhbs-jaas.2DL-1*		-2.05 (E3/9.86) =	-2.02 (E3/9.18) =
	*QFhbs-jaas.4AL-1*			-1.38 (E2/5.36) =
	*QFhbs-jaas.6AS-1*			

*^*a*^FHB|PH: FHB without the influence of PH; FHB|SC: FHB without the influence of SC; FHB|FD: FHB without the influence of FD. Numerals before the parentheses are estimates of the additive effect of the conditional QTL. E and numerals in parentheses indicate the environment in which the QTL was detected (E1: Yangzhou 2015, E2: Yangzhou 2016, E3: Yangzhou 2017) and the percentage of phenotypic variance explained by the additive effects of the mapped QTL, respectively. “-” or “+” following the parentheses denotes a conditional QTL with an increase or decrease in PVE% to that of the corresponding traditional QTL, respectively. “=”, is placed after the parentheses to denote a conditional QTL with equal PVE% to that of the traditional QTL. The following letter “b” denotes a conditional QTL with a similar location in traditional QTL mapping analysis but detected in a different environment.*

After spray inoculation in E2 and E3, four, three, and one of the six putative traditional FHB QTL with respect to PH, SC, and FD were unmapped, respectively (**Table 4**). *QFhbs-jaas.1AS* was identified in E2 when conditioned on FD with a reduced contribution and was also detected in E3 when conditioned on PH and FD. *QFhbs-jaas.1AL* was detected with reduced and equal contributions in E2 or E3, when FHB was conditioned on the three related traits. *QFhbs-jaas.2AL* showed equal contributions by a conditional QTL mapping analysis when conditioned on FD, and while whereas a decreased contribution in E3 when conditioned on SC. Excluding the influence of SC and FD on FHB, *QFhbs-jaas.2DL-1* exhibited equal contributions in E3 to those of traditional QTL. *QFhbs-jaas.4AL-1* explained 5.36% of the phenotypic variation when FHB was conditioned on FD in E2 and showed a contribution equal to that of the corresponding traditional QTL.

Two and three additional QTL for FHB conditioned on SC after point and spray inoculation, were detected only by conditional QTL mapping analysis, respectively (**Table 5** and **Figure 2**). Among the five additional conditional QTL, three were detected with negative additive values, indicating that the favorable alleles were from the FHB-resistant parent C615.

**Table 5:** Extra conditional QTL for FHB resistance with respect to the related agronomic traits in the C615/Yangmai 13 RIL population.

Trait^a^	Extra conditional QTL	Env.^b^	Closest marker	Position interval (cM)	LOD value	PVE%	Additive effect
FHB point|SC	*QFhbp|Sc-jaas.1AS*	E1	BobWAite_c12568_900	40.0-47.0	2.53	4.55	-0.35
	*QFhbp|Sc-jaas.7BL-1*	E3	Excalibur_c61073_128	60.0-70.1	3.55	6.2	-0.56
FHB spray|SC	*QFhbs|Sc-jaas.3AL*	E3	wsnp_Ex_c5047_8963671	42.9-53.0	2.73	4.92	1.05
	*QFhbs|Sc-jaas.6DS-1*	E2	BobWAite_c15802_72	0.0-12.9	2.65	4.92	1.52
	*QFhbs|Sc-jaas.7BL-1*	E3	GENE-4710_573	109.7-116.4	3.77	11.95	-1.85

*^*a*^See footnotes to **Table 1** for abbreviations. ^*b*^E1: Yangzhou 2015, E2: Yangzhou 2016, E3: Yangzhou 2017.*

## Discussion

### Linkage Map Construction

High-density linkage maps are required for genetic studies on common wheat and its large, complex genome (Peleg et al., 2008; Zhai et al., 2016). Single nucleotide polymorphisms (SNPs) that are widely distributed throughout the genome have been used in QTL mapping (McCartney et al., 2016; Cui et al., 2017). In the current study, QTL mapping was conducted using the wheat 90K chip (Wang et al., 2014), and 6,321 polymorphic SNPs markers were allocated to 34 LGs. One reason for the high number of LGs is that the construction of this linkage map adopted a high LOD (LOD > 10). Another reason might be that some chromosome segments in bi-parents consisted of numerous monomorphic SNPs, and the original SNPs in the 90K chip were unevenly distributed among chromosomes. Comparable results were reported by Giancaspro et al. (2016) and He et al. (2016). Among these polymorphic SNPs, 650 SNPs (16.7%) were located in the D genome (especially on 4D, 5D, 6D, and 7D), much less abundant than those for the A (46.4%) and B (36.9%) genomes (Supplementary Tables S2, S3). The low coverage of the D genome was in agreement with previous reports (Wu et al., 2015; Zou et al., 2016).

### Traditional and Conditional QTL for FHB Resistance

In this study, conditional QTL mapping was used to analyze FHB response conditioned on related agronomic traits, including PH, SC, and FD at the individual QTL level. The conditional QTL could be divided into four types by comparing the genetic effects of traditional QTL (Cui et al., 2011; Li et al., 2015). When our analysis of QTL for FHB resistance conditioned on SC, four different outcomes are generated: (1) a QTL was (for example, *QFhbp-jaas.4AL-1* in E1 and E2) detected only in traditional QTL analysis, indicating that this QTL is completely influenced by SC; (2) a QTL (for example, *QFhbs-jaas.2DL-1* in E3) was identified both in traditional and conditional QTL analysis with a very similar effect, showing that this QTL only improves FHB resistance and is not influenced by SC; (3) a QTL for FHB resistance showed either a greatly reduced or enhanced effect, implying that this QTL is partly influenced by SC. For example, *QFhbp-jaas.2DS-1* exhibited enhanced effects in E1 and E2; (4) an “extra” QTL (for example, *QFhbp|Sc-jaas.7BL-1* in E3) for FHB resistance was detected only in conditional QTL analysis, indicating that this QTL is completely masked by SC. Hence, the extra QTL was detected by its effect on FHB response, but has an opposite effect from SC.

Among the seven traditional QTL for FHB resistance detected following point inoculation, four, two, and one were completely, partly, and partly or not influenced by SC, respectively (**Table 4**). However, after spray inoculation, four, three, and one of six traditional QTL were completely influenced by PH, SC, and FD, respectively; and zero, zero, and three were independent of PH, SC, and FD (**Table 4**). At the QTL level, these results indicated that SC strongly influences FHB resistance after point inoculation. However, after spray inoculation, the FHB response was affected by the three agronomic traits and both PH and SC had greater contributions to FHB resistance than did FD. This was probably due to the fact that the interior of one spike was injected with a fixed amount of inoculum. Similar results were also found in correlation analysis between FHB response and agronomic traits (**Table 2**). To date, some hypotheses have been reported to explain these relationships (Klahr et al., 2007; Lu et al., 2011; Giancaspro et al., 2016; Malihipour et al., 2016). In general, spikes of higher lines may dry faster and be less infected by disease. The earlier- or later-flowering lines may be in the special environments (e.g., low humidity and temperature) that are not suitable for disease development. However, these traits are very undesirable in wheat breeding. Using the conditional QTL analysis to evaluate their relationships at the QTL level, we may resolve this contradiction through selecting appropriate resistance QTL. We also found that five additional conditional QTL for FHB resistance were entirely suppressed by SC (**Table 5**). These findings suggest that conditional QTL analysis reduce confounding QTL and other traits and thereby facilitates in the elucidation of the genetic mechanism underlying FHB resistance.

### Comparison to Previous Studies

Thirteen QTL for FHB resistance were identified in this study (**Table 3** and **Figure 2**). These QTL mainly from C615, but a few were from Yangmai 13 and accounted for low proportion of phenotypic variance (<12%). This low phenotypic variance might be affected by the quality of inoculation and the environments (Buerstmayr et al., 2009; He et al., 2016; Zhu et al., 2016). Among these, the resistance QTL on chromosome 2AL, 2DS, and 2DL were detected in both traditional and conditional QTL mapping, respectively.

The major QTL on chromosome 2DL was detected in both point and spray inoculated experiments across 3 years; this QTL was also closely linked to the SNP marker *TA001163-0861*. He et al. (2016) detected a QTL for FHB resistance on chromosome 2DL from CIMMYT line Soru#1, and in that case the QTL was in the marker interval *Kukri_c36639_186*–*Xgwm539* and explained 14–20% of the phenotypic variation. SNP markers *TA001163-0861* and *Kukri_c36639_186* were on the 90K consensus map with a genetic distance of 5.4 cM corresponding to a physical interval of 21 Mb in the Chinese Spring RefSeq v1.0 sequence (Wang et al., 2014). In addition, Lu et al. (2013) identified a QTL on chromosome 2DL for Type I and Type II resistance from Chinese line Shanghai-3; here the QTL was tightly linked to SSR marker *Xgwm539*. Considering that both C615 and Soru#1 have Shanghai-3 in their pedigrees, these two QTL are likely the same. Additionally, this QTL that is responsible for different types of resistance to FHB has been detected in several other FHB-resistant germplasms, including Wuhan-1 (Somers et al., 2003), Wangshuibai (Lin et al., 2004), CJ9306 (Jiang et al., 2007), VA00W-38 (Liu et al., 2012), and SYN1 (Zhu et al., 2016). These reports collectively indicate that this QTL might be an important “true QTL”, with great potential for marker-assisted selection.

The QTL on chromosome 2AL found after spray inoculation was linked to SNP marker *BS00022896_51* and explained 5.23–9.34% of the phenotypic variation. Three QTL clusters for FHB resistance were previously detected on chromosomes 2AS (2) and 2AL, and these were closely linked to SSR markers *Xbarc124, Xgwm122*, and *Xgwm311*, respectively (Gervais et al., 2003; Ma et al., 2006; Holzapfel et al., 2008; Lu et al., 2013; Zhang et al., 2014; Giancaspro et al., 2016; Petersen et al., 2016, 2017). Markers *Xgwm311* (at 772 Mb) and *BS00022896_51* (at 612 Mb), separated by a physical distance of 160 Mb, are in different deletion bins (Qi et al., 2004). Hence, *QFhbs-jaas.2AL* is likely a novel QTL. To our knowledge, only one QTL on chromosome 2DS was found in previous studies (Liu et al., 2009; Buerstmayr et al., 2011; Cai and Bai, 2014; McCartney et al., 2016) and was closely linked to the SSR marker *Xgwm261* (close to *Rht8*) at about 20 Mb. Here, *QFhbp-jaas.2DS-1* for Type II resistance was closely linked to the SNP marker *Kukri_c60627_74* at 74 Mb. The SSR and SNP markers were separated by a physical distance of 44 Mb. This QTL was in a similar position to a QTL previously reported for anther extrusion in a German cultivar (He et al., 2016), which was not linked to marker *Xgwm261*. Thus, *QFhbp-jaas.2DS-1* is likely to be new, but these two resistance QTL should be verified by future work.

### Implications for Wheat Breeding

Resistance to FHB in wheat is a complex trait and marker-assisted selection is a valuable tool to improve FHB resistance. In the present study, the three stable resistance QTL from C615 on chromosomes 2AL, 2DS, and 2DL reported here belong to the second or third type of conditional QTL. These QTL were partly affected by or were independent of three related agronomic traits, and thus should be given more consideration for developing resistant varieties with good agronomic traits. Indications are that when the three resistance QTL are combined, these can reduce susceptibility by 30–40% (Supplementary Table S4). These SNP markers closely linked to the QTL detected here may be effectively used in marker-assisted selection for improving FHB resistance when C615 is used as resistance donor. The results of this study also indicate that conditional QTL mapping analysis can improve our understanding of complex traits.

## Ethics Statement

We declare that these experiments comply with the ethical standards in China.

## Author Contributions

YZ and SCheng conceived and designed the experiments. XY and JC performed the experiments. XY and ZJ analyzed the data. WH, TB, DG, DL, RW, YL, SChen, XC, and JL contributed reagents, materials, or analysis tools. XY wrote the manuscript. All authors read and approved the final manuscript.

## Conflict of Interest Statement

The authors declare that the research was conducted in the absence of any commercial or financial relationships that could be construed as a potential conflict of interest.

## Footnotes

1 http://www.earthatlas.mapchart.com

2 http://statgen.ncsu.edu/qtlcart/WQTLCart.htm

3 http://ibi.zju.edu.cn/software/qga/
