# Supplementary material for: Genetic Analysis of Fusarium Head Blight Resistance in CIMMYT Bread Wheat Line C615 Using Traditional and Conditional QTL Mapping
Source: Front Plant Sci. 2018 May 1;9:573. doi: 10.3389/fpls.2018.00573 (PMC5946024; doi:10.3389/fpls.2018.00573)
Supplement: Supplementary file 2 [file Image_1.PDF]

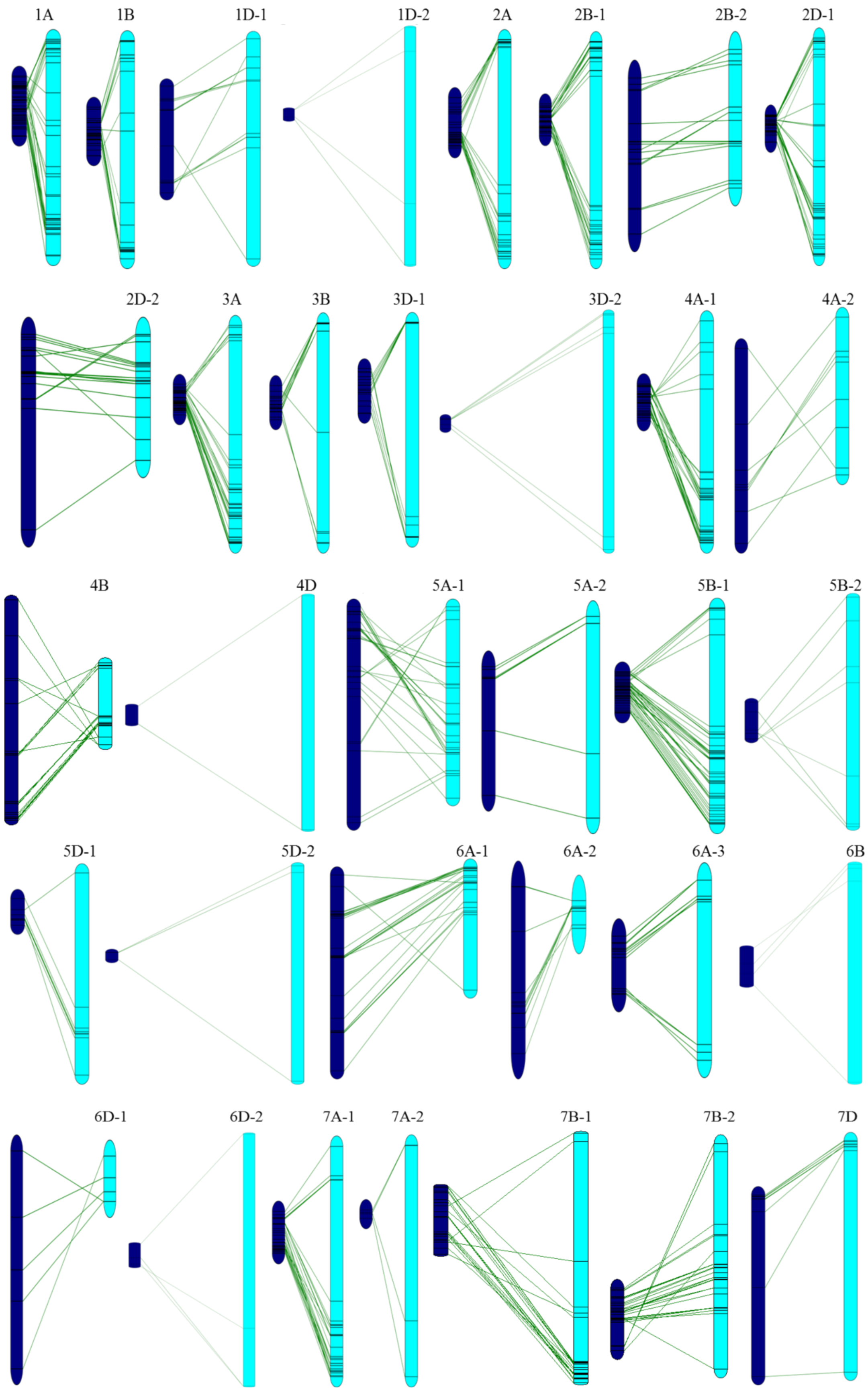

Figure S1. Synteny studies of the SNPs from the linkage map (in blue) and the wheat reference genome (in green)
